# Current landscape of academic and point of care CAR-T cell therapy in low-middle income countries

**DOI:** 10.46989/001c.163505

**Published:** 2026-06-29

**Authors:** Sofía I. Quezada-Ramírez, José E. Montelongo-Cepeda, Gabriel A. González-López, Rosario Salazar-Riojas, Dalila M. Alvarado-Navarro, Andrés Gómez-De León, David Gómez-Almaguer

**Affiliations:** 1 Hematology Service, Hospital Universitario “Dr. José Eleuterio González”, Universidad Autónoma de Nuevo León, Monterrey, México

**Keywords:** CAR-T, lower-income countries, cellular therapy, point-of-care

## Abstract

Chimeric antigen receptor T-cell (CAR-T) therapy has emerged as a transformative treatment for patients with relapsed or refractory hematologic malignancies, offering new possibilities beyond conventional transplantation. Since their approval in high-income countries, CAR-T products have demonstrated remarkable efficacy; however, their implementation in low- and middle-income countries (LMICs) remains limited due insufficient infrastructure, complex regulatory requirements and high costs. In some countries, like Mexico, these barriers are particularly pronounced, with limited access to clinical-grade vectors, early phase clinical research programs, and lack of government and private sector infrastructure and funding opportunities. Facing these challenges, there is a substantial patient population with B-cell acute lymphoblastic leukemia, multiple myeloma, or diffuse large B-cell lymphoma, who could benefit from CAR-T therapy. Advances in point-of-care manufacturing within academic settings –particularly the use of closed, semi-automated systems– have demonstrated feasibility in LMICs, by reducing costs and simplifying procedures, while maintaining product quality. These approaches may provide a viable alternative to commercial products, with the potential to lower the economic burden and increase accessibility. Regulatory innovation with the establishment of expert multidisciplinary oversight committees, will also be critical to ensure safe implementation. Ultimately, CAR-T therapy in LMICs is both a scientific and public health opportunity, and its integration into healthcare systems will depend on collaborative strategies that address financial, logistical, and policy barriers. By highlighting our experience, this review underscores the importance of developing locally adapted solutions to expand access to advanced cellular therapies in resource-constrained settings.

## Introduction

Chimeric antigen receptor T cells (CAR-T cells) are a novel therapy that has revolutionized the management of patients with relapsed or refractory B-cell derived hematological malignancies.^1,2^ The first CAR-T therapy for acute lymphoblastic leukemia (ALL) was approved for commercialization in the United States and the European Union in 2017 and 2018, respectively.^3,4^ Nonetheless, health systems in lower and middle-income countries (LMICs) have not been able to adopt this line of therapy in their standard-of-care (SOC) due to multiple factors, including regulatory barriers, lack of infrastructure, high costs, and inexperience. Among these barriers, high cost emerges as the common denominator across different resource settings; even in the United States, access to this therapy remains largely constrained by the limited coverage provided by insurance systems.^5–9^ Approximately 70% of FACT/JACIE-accredited centers which manufacture CAR-T cells face significant challenges related to production costs and regulatory requirements. These difficulties appear to be even more pronounced in centers located outside the United States.^10^ In our region, Brazil remains the only country in Latin America (LatAm) with an established regulatory framework with commercial CAR-T product approvals for clinical use.^11^ In LatAm, efforts across multiple institutions are ongoing including in Brazil, Chile, Colombia, Mexico, and Uruguay.^12^ In this brief review, we explore the role of CAR-T cells in LMICs and the main limitations which prevent their availability.

## Target populations

Currently, commercially available CAR-T cell therapies are approved for B-cell neoplasms such as B-cell acute lymphoblastic leukemia (B-ALL),^13^ non-Hodgkin lymphoma (NHL), and multiple myeloma (MM) (see Table 1). According to the 2021 Global Burden of Disease (GBD) study, the annual incidence rates in Mexico are 5.71 per 100,000 inhabitants for NHL, 2.36 for ALL, and 1.56 for multiple myeloma (MM) – figures comparable to those reported across Latin America and the Caribbean.^14^ Nationwide data on relapse or refractory (R/R) disease are limited, but the Mexican Acute Leukemia Workgroup (GTLA) has documented a relapse rate of 62% among Mexican adults with ALL.^15^ In addition, Mexican patients with LNH and MM tend to be younger than their counterparts worldwide, which could make them more suitable candidates for CAR-T therapy.^16,17^ Notably, 30-40% patients with diffuse large B-cell lymphoma (DLBCL), the most common NHL subtype, relapse after first-line therapy^18^ and about 20% relapse even after autologous stem cell transplantation (auto-HCT).^19^ Similarly, a retrospective study of patients within a Mexican healthcare system revealed that, despite treatment with novel agents, relapse of MM occurred in 50% of evaluable patients during the follow-up period.^20^ Because relapse after treatment carries a poor prognosis, CAR-T cell therapy could play a crucial role in this younger population.

**Table 1.** Approved commercial CAR-T products in the United States, European Union, and Latin America.

**Regulatory agency**	**Countries**	**Product**	**Date of first approval**	**Indication(s)**
Food and Drug Administration (FDA)	United States of America	Idecabtagene Vicleucel^21^	March, 2021	R/R MM after ≥2 lines of therapy
		Obecabtagene Autoleucel^22^	November, 2024	Adults with R/R B-ALL
		Lisocabtagene Maraleucel^23^	June, 2022	Adults with large B-cell lymphoma and advanced follicular lymphoma
		Ciltacabtagene Autoleucel^24^	February, 2022	R/R MM with ≥1 line of therapy
		Tisagenlecleucel^3^	August, 2017	Children and young adults (<26 yo) with B-ALL if refractory or after 2nd relapse Adults with R/R large B-cell lymphoma or R/R follicular lymphoma after 2 lines of therapy
		Brexucabtagene Autoleucel^25^	July, 2020	Adults with R/R B-ALL or mantle cell lymphoma
		Axicabtagene Ciloleucel^26^	October, 2017	Adults with R/R B-ALL or with R/R large B-cell lymphoma
European Medicines Agency (EMA)	European Union Member States, Iceland, Norway, and Liechtenstein	Axicabtagene Ciloleucel^27^	August, 2018	Adults with R/R high-grade B-cell lymphoma (HGBL), diffuse large B-cell lymphoma (DLBCL), primary mediastinal large B-cell lymphoma (PMBCL) after ≥2 lines of therapy. Adults with R/R follicular lymphoma after ≥3 lines of therapy.
		Brexucabtagene Autoleucel^28^	December, 2020	Adults with R/R mantle cell lymphoma after ≥2 lines of therapy and adults (>25 yo) with R/R B-ALL
		Tisagenlecleucel^29^	August, 2018	Children and young adults (<26 yo) with R/R B-ALL that is either refractory, have relapsed after HCT or after ≥2nd relapse Adults with R/R diffuse large B-cell lymphoma (DLBCL) after ≥2 lines of therapy Adults with R/R follicular lymphoma after ≥2 lines of therapy
		Ciltacabtagene Autoleucel^30^	May, 2022	Adults with R/R MM after ≥1 line of therapy
		Lisocabtagene Maraleucel^31^	April, 2022	Adults with R/R high-grade B-cell lymphoma (HGBL), diffuse large B-cell lymphoma (DLBCL), primary mediastinal large B-cell lymphoma (PMBCL), or grade 3B follicular lymphoma. Adults with R/R high-grade B-cell lymphoma (HGBL), diffuse large B-cell lymphoma (DLBCL), primary mediastinal large B-cell lymphoma (PMBCL). Adults with R/R follicular lymphoma after ≥2 lines of therapy.
		Idecabtagene Vicleucel^32^	August, 2021	Adults with R/R MM after ≥2 lines of therapy
		Obecabtagene Autoleucel^33^	July, 2025	Adults (>25 yo) with R/R B-ALL.
National Health Surveillance Agency (ANVISA)	Brazil	Tisagenlecleucel^34^	February, 2022	Pediatric and young adult patients ≤25 years of age with R/R B-ALL Adult patients with R/R diffuse large B-cell lymphoma (DLBCL) Adult patients with R/R follicular lymphoma (FL)
		Ciltacabtagene Autoleucel^35^	March, 2022	Adult patients with R/R MM Adult patients with MM who have previously received a proteasome inhibitor and are refractory to lenalidomide.
		Axicabtagene Ciloleucel^36^	August, 2023	Adult patients with B-cell lymphoma refractory to first-line chemoimmunotherapy or relapsed within 12 months of first-line chemoimmunotherapy. Adult patients with relapsed or refractory large B-cell lymphoma (LBCL) after two or more lines of systemic therapy, including diffuse large B-cell lymphoma (DLBCL) not otherwise specified, primary mediastinal large B-cell lymphoma, high-grade B-cell lymphoma (HLBCG), and DLBCL arising from follicular lymphoma.
		Brexucabtagene Autoleucel^37^	December, 2023	Adults with R/R Mantle Cell Lymphoma (MCL) Patients with R/R B-cell precursor Acute Lymphoblastic Leukemia (ALL)

B-ALL = B acute lymphocytic leukemia; R/R = relapsed or refractory; MM = multiple myeloma

Additionally, CAR-T therapy may have a role in severe autoimmune conditions. A German group has reported success on selected autoimmune patient cases, such as systemic lupus erythematosus (SLE).^38^ Although not yet approved for this indication, these special patient groups will increase the demand for CAR-T products and may incentivize POC development in the near future.^39^

## Role of CAR-T cells in the context of transplant

CAR-T and HCT represent transformative therapies that can alter the course of patients with hematological malignancies, and current evidence suggests that they may be complementary rather than strictly competitive.^40^ Previous transplant status has not been shown to impact CAR-T efficacy.^41^ In NHL, patients experiencing relapse beyond 12 months are generally managed with auto-HCT, whereas those with primary refractory disease or early relapse are more likely to benefit from CAR-T therapy.^42^ In pediatric ALL, relapse after HCT remains the leading cause of treatment failure, affecting up to 15-20% of cases, with subsequent management individualized due to lack of standardized algorithms.^43,44^ In this setting, CAR-T therapy has emerged as a promising option, and ongoing studies are evaluating its optimal positioning in relation to HCT. In adults with ALL, early data indicate that CAR-T followed by consolidation with a second allo-HCT improves long-term survival in transplant-refractory cases; however, whether this approach offers comparable benefits in pediatric patients remains to be determined.^44^

Unlike transplantation, CAR-T does not require identification of a compatible donor, thereby reducing the time from relapse to treatment. This is particularly relevant for Hispanic populations, in whom genetic heterogeneity poses challenges to donor matching. For example, in a recent cohort of Hispanic patients in the United States who were candidates for transplant, only 47% were able to identify an 8/8 HLA-matched donor.^45^ Although not innocuous, CAR-T therapy circumvents transplant-related complications such as immunosuppression, graft-versus-host disease (GvHD), transmission of infectious diseases and other procedure-associated toxicities, while offering comparable response rates.^46^ These toxicities are traded for complications such as cytokine release syndrome (CRS), immune-effector cell neurotoxicity syndrome (ICANS), long-term cytopenias, secondary CAR-T derived malignancies, and the possibility of disease relapse.^47^

Nevertheless, advances in transplantation have also expanded access and improved outcomes. The introduction of post-transplant cyclophosphamide (PTCy) has markedly improved haploidentical transplant results.^48^ In a multinational cohort, 100% of Hispanic patients were able to identify haploidentical donors with 5/8 HLA matches.^45^ Haploidentical transplantation has increased feasibility in many low- and middle-income countries (LMICs), offering greater donor availability and lower costs compared to unrelated matched donors.^49,50^ However, as will be discussed in subsequent sections, CAR-T therapy may offer an additional cost advantage, particularly with the emergence of point-of-care (POC) platforms, which can produce cost-effective individual products appraised at approximately $34,000 USD.^51^ An individualized case-by-case choice must be made for patients in LMICs, according to their performance status, insurance availability, and disease burden.

## Point-of-care manufacturing

Centralized manufacturing requires centers to incur in expenses related to cryopreservation, logistically complex transportation and, simultaneously, may implicate prolonged turnaround times, reduced flexibility in urgent clinical scenarios and, with commercial products, higher costs for patients and payors. However, it can be simpler from a regulatory oversight perspective and cost-effective for large manufacturers. Point-of-care (POC) manufacturing – in which CAR-T production is performed within or adjacent to the hospital where the patient is treated – provides reduced costs, shorter turnaround times, and improved accessibility. Therefore, it is attractive to incorporate POC manufacturing in universities or research-based Good Manufacturing Practices (GMP) facilities (academic manufacturing), to care for their own patients, but also to potentially serve multiple hospitals, even in a regional or national framework, particularly when lacking a centralized alternative. Nonetheless, POC manufacturing also requires a heavy investment for institutions on the establishment of GMP-compliant clean rooms, specialized supplies, and training of expert laboratory staff that can lead to successful validation and implementations. Despite these challenges, the POC/academic approach has demonstrated viability and reproducibility across multiple centers and products, achieving reduced CAR-T cell manufacturing times, and enabling treatment of patients with rapidly progressing disease.^52^ Promising feasibility results from phase 1 and 2 studies in acute leukemias have been described with different POC strategies by Israeli teams. Notably, no manufacturing failures were reported, whilst maintaining high early remission rates.^53,54^ Long-term follow-up will be needed. However, with the currently available data, there does not appear to be significant differences between POC and commercial CAR-T products.

Our institution has begun the process of establishing a decentralized CAR-T manufacturing facility. In 2024, we successfully manufactured five second-generation anti-CD19 CAR-T products from healthy volunteer donors with the *CliniMACS Prodigy* closed system. Our approach resulted in high transduction and cell viability rates with sterility maintained throughout the process,^51^ comparable to industrial-grade products and to the results from other institutions using the same vector. Although facilitated by our center’s experience and resources for HCT, our CAR-T manufacturing strategy required a hefty investment (approx. $816,000 USD), including the purchase of the *CliniMACS Prodigy,* building a clean-room, consumable supplies, and personnel training. Using the same closed system, an academic initiative in Spain has successfully achieved regulatory approval by the national agency, allowing for commercial use of *varnimcabtage autoleucel*, a CD19 CAR-T cell product manufactured in multiple centers.^55^ Currently, our institution is seeking approval from the national regulatory agency COFEPRIS to conduct a phase I/II clinical trial with the anti-CD19 CAR-T produced in our center.

Additionally, recent successes in early-phase studies^56^ suggest that *in vivo* CAR-T therapies may represent an alternative strategy to reduce manufacturing-related costs, as the aforementioned infrastructure could potentially be omitted. Nevertheless, extending this success to LMICs is likely to be time-consuming, as *in vivo* CAR-T approaches would still require individual national regulatory approval, and a robust lentiviral transportation and storage infrastructure would remain necessary.

### Closed systems in academic context

Closed, semi-automated systems reduce the risk of contamination and minimize operator-dependent errors.^57^ These systems help preserve product quality while lowering manufacturing costs, resulting in up to 75% reduction in failure rates.^58^ Consequently, academic centers increasingly favor closed, semi-automated platforms, with Miltenyi’s *CliniMACS Prodigy* and Lonza’s *Cocoon* being among the most commonly used.^10^ *CliniMACS Prodigy* (Miltenyi Biotec, Bergisch Gladbach) integrates a cell culture unit capable of selection, washing and automated expansion of cells.^59^

Comparable initiatives in other LMICs further underscore its feasibility. In India, the same platform demonstrated consistent efficacy throughout the manufacturing process.^60^ In 2020, a Brazilian academic group reported the capacity to manufacture CAR-T cells with potent cytotoxicity and specificity against CD19+ targets.^61^ A subsequent study has also demonstrated the clinical efficacy and safety of academic CD19 CAR-T cells in patients with R/R Hodgkin’s lymphoma and ALL.^62^ Other examples of successful manufacturing utilizing the *CliniMACS Prodigy* platform include teams from Thailand^63^ and Kazakhstan.^64^

## Regulatory frameworks in LATAM/LMICs

A major challenge in LMICs is the lack of a regulatory framework for advanced cell therapies. National regulatory agencies may lack experience, training, funding and time to develop the necessary requirements, guide the legal modifications to effectively evaluate commercialization requests and the clinical trial approval for CAR-T products and others. Therefore, a key step is the establishment of dedicated, semi-autonomous committees within the national healthcare authorities, composed of experts in advanced cellular therapies. A review of national healthcare authorities in the United States, the European Union, and several Latin American countries reveals a heterogenous landscape, with some establishing dedicated internal bodies for the assessment and approval of these complex therapies (see Table 2).

**Table 2.** National healthcare authorities and the presence of dedicated internal organisms for the regulation of advanced cellular therapies in selected regions

**Country/Region**	**National Healthcare Authority**	**Dedicated Internal Organism**
Argentina	*Administración Nacional de Medicamentos, Alimentos y Tecnología Médica* (ANMAT)	None
Brazil	*Agência Nacional de Vigilância Sanitária* (ANVISA)	Advanced Therapy Technical Committee
Chile	*Instituto de Salud Pública* (ISP)	Specialized commission for the evaluation of advanced therapies
Colombia	*Instituto Nacional de Vigilancia de Medicamentos y Alimentos* (INVIMA)	None
European Union Member States, Iceland, Norway, and Liechtenstein	European Medicines Agency (EMA)	Committee for Advanced Therapies (CAT)
Mexico	*Comisión Federal para la Protección contra Riesgos Sanitarios* (COFEPRIS)	None✝
Peru	*Dirección General de Medicamentos, Insumos y Drogas* (DIGEMID)	None
United States of America	Food and Drug Administration (FDA)	Office of Therapeutic Products (OTP) within the Center for Biologics Evaluation and Research (CBER)

✝ In 2023, COFEPRIS started to assemble a small group of experts to create an initial regulatory framework for advanced cellular therapies. Nonetheless, it did not establish a formal dedicated committee or organism for this purpose.^12^

In Mexico, the regulatory framework for cellular therapies remains at an early stage of development. The Mexican Health Regulatory Agency (COFEPRIS) is forming an expert committee with the aim of formulating specific recommendations for modifying existing regulation and legislation, and to define the necessity and characteristics of novel regulatory processes, requirements and documentation.^12^ This in the context of a lack of international harmonization and variations in the regulatory framework. From the perspective of investigators, the current environment has resulted in prolonged timelines for review and approval, with limited mechanisms available to provide guidance and to support institutions and investigators seeking authorization for new therapies. Furthermore, the establishment of clear GMPs and regulatory frameworks for the procurement of health-related supplies that are inclusive and supportive of academic efforts would considerably facilitate this process.

## Costs

The manufacturing and administration of CAR-T therapies represent a substantial cost for healthcare.^65^ In addition to direct product expenses, there are multiple hidden costs associated with CAR-T implementation, including infrastructure requirements, regulatory certifications, specialized staff, supportive therapies and post-infusion care.

Currently, pharmaceutical companies manufacture CAR-T cells through centralized systems, which are efficient at scale but result in long patient waiting times and substantial logistical challenges. While this model reduces production costs, the expenses associated with distribution remain high, and the commercial prices set by some companies render these therapies inaccessible to most of the global population. In contrast, POC manufacturing offers notable advantages, such as shorter production times and lower costs, contributing to greater accessibility.^60^ However, this approach requires highly trained personnel, specialized clinical centers for patient care and robust regulatory oversight.^55^

Across Latin America, several countries such as Colombia, Brazil, Chile, and Uruguay have also begun to establish CAR-T cell programs. In Mexico, multiple initiatives focused on local production and clinical application of CAR-T cells are currently being developed by various institutions, including our center, the National Institute of Medical Sciences and Nutrition “Salvador Zubirán”, and the National Institute of Pediatrics, among others. Recently, the Mexican government has proposed the establishment of centralized CAR-T cell manufacturing in collaboration with public institutions and private sector support. This initiative aims to reduce production costs and establish a centralized network to facilitate the distribution of CAR-T products to various centers across the country.^66^ Additionally, the partnership between the Oswaldo Cruz Foundation (Fiocruz) and Caring Cross seeks to develop local manufacturing capacity for CAR-T cells and lentiviral vectors, with the goal of facilitating access in Brazil and Latin America.^67^

With regard to the total cost of care, encompassing patient evaluation through post-infusion management, the average Medicare expenditure for inpatient CAR-T therapy in the United States was reported at $498,723 USD while the outpatient setting averaged $414,393 USD.^68^ In Portugal, the median cost per treated patient was €355,195, with the CAR-T accounting for 97% of the total expense.^69^ To our knowledge, no comprehensive data are currently available regarding the overall cost of care in Latin America. Focusing exclusively on cell production, our center achieved CAR-T cells generation at USD $32,073.29, a figure consistent with that reported in India ($35,107 USD), representing a nearly 90% reduction, compared with the manufacturing costs in the United States.^51,60^

## Conclusions

CAR-T cell therapy represents a transformative treatment for R/R hematologic malignancies. Yet, its adoption in LMICs such as Mexico remains limited by infrastructural, regulatory and financial barriers. Despite these challenges, growing experience with POC and academic manufacturing through the use of closed production systems have demonstrated that locally adapted, cost-effective solutions are feasible as a starting point. Strengthening regulatory capacity through specialized committees will be essential to ensure safe and equitable access. Ultimately, CAR-T therapy in LMICs is not only a scientific opportunity but also a healthcare imperative to address the needs of a high-burden patient population.

### Conflicts Of Interests

AGDL: Honoraria for lectures: J&J, Sanofi, Amgen, BMS, Astellas; Advisory board: Pfizer, MSD, J&J. DGA: Speaker/Advisory: Amgen, Roche, Novartis, Takeda, Janssen, BMS, Sanofi, Abbvie, Teva, Astra, Asofarma.

The rest of the authors declare they have no conflicts of interest to disclose.

### Authors’ Contribution

Conceptualization: Sofía I. Quezada-Ramírez, José Emiliano Montelongo-Cepeda, Gabriel A. González-López, David Gómez-Almaguer.

Formal Analysis: Sofía I. Quezada-Ramírez, José Emiliano Montelongo-Cepeda, Gabriel A. González-López.

Methodology: David Gómez-Almaguer, Sofía I. Quezada-Ramírez, José Emiliano Montelongo-Cepeda, Gabriel A. González-López.

Supervision: Rosario Salazar-Riojas, Dalila M. Alvarado-Navarro, Andrés Gómez-De León, David Gómez-Almaguer.

Writing – original draft: Sofía I. Quezada-Ramírez, José Emiliano Montelongo-Cepeda, Gabriel A. González-López.

Writing – review & editing: Rosario Salazar-Riojas, Dalila M. Alvarado-Navarro, Andrés Gómez-De León.

All authors have confirmed this manuscript for publication.

### Ethical Conduct Approval

This article is a review of previously published literature and does not involve human participants, animals, or identifiable patient data. Therefore, Institutional Review Board (IRB) approval and ethical committee approval were not required.

### Informed Consent Statement

Not applicable. This study is a review of previously published literature and does not involve human participants or patient data.

## Data Availability

Data sharing is not applicable to this article as no new data were generated or analyzed in this study.
